# The fatty acid binding protein 7 (FABP7) is involved in proliferation and invasion of melanoma cells

**DOI:** 10.1186/1471-2407-8-276

**Published:** 2008-09-30

**Authors:** Ana Slipicevic, Kjersti Jørgensen, Martina Skrede, Anne Katrine Ree Rosnes, Gunhild Trøen, Ben Davidson, Vivi Ann Flørenes

**Affiliations:** 1 Pathology Clinic, Rikshospitalet-Radiumhospitalet Medical Center, Montebello N-0310 Oslo, Norway; 2 Faculty Division Radiumhospitalet, Medical Faculty, University of Oslo, Oslo, Norway

## Abstract

**Background:**

The molecular mechanisms underlying melanoma tumor development and progression are still not completely understood. One of the new candidates that emerged from a recent gene expression profiling study is *fatty acid-binding protein 7 *(*FABP7)*, involved in lipid metabolism, gene regulation, cell growth and differentiation.

**Methods:**

We studied the functional role of FABP7 in human melanoma cell lines and using immunohistochemistry analyzed its expression pattern and clinical role in 11 nevi, 149 primary melanomas and 68 metastases.

**Results:**

FABP7 mRNA and protein level is down-regulated following treatment of melanoma cell lines with a PKC activator (PMA) or MEK1 inhibitor (PD98059). Down-regulation of FABP7 using siRNA decreased cell proliferation and invasion but did not affect apoptosis. In clinical specimens, FABP7 was expressed in 91% of nevi, 71% of primary melanomas and 70% of metastases, with a cytoplasmic and/or nuclear localization. FABP7 expression was associated with tumor thickness in superficial spreading melanoma (P = 0.021). In addition, we observed a trend for an association between FABP7 expression and Ki-67 score (P = 0.070) and shorter relapse-free survival (P = 0.069) in this group of patients.

**Conclusion:**

Our data suggest that FABP7 can be regulated by PKC and the MAPK/ERK1/2 pathway through independent mechanisms in melanoma cell lines. Furthermore, FABP7 is involved in cell proliferation and invasion *in vitro*, and may be associated with tumor progression in melanoma.

## Background

Malignant melanoma is the most lethal skin cancer and accounts for about 75% of all deaths from skin tumors. In its early stage, melanomas can be treated surgically, but once the tumor has progressed, it is difficult to treat and it does not respond to current therapies. The molecular mechanisms underlying melanoma development and progression are still not completely understood, and novel diagnostic and prognostic markers, as well as therapeutic targets are needed [1].

We have previously reported that phorbol-12-myristate-13-acetate (PMA), a protein kinase C (PKC) activator, increases proliferation and promotes anchorage-independent survival of melanoma cells cultivated as multicellular aggregates in suspension (spheroids). This protective action was at least partly mediated through PKC and MEK-independent activation of the mitogen-activated protein kinase/extracellular signal-regulated kinases 1/2 (MAPK/ERK1/2) [2]. In an attempt to identify additional genes involved in survival and apoptosis of melanoma cells, we used high throughput gene expression profiling (Affymetrix™) to identify differentially expressed genes in untreated cells cultured as monolayer or spheroids, as well as in spheroids treated with PMA and/or the MEK1 inhibitor PD98059. The analysis revealed that the *fatty acid-binding protein 7 (FABP7, BLBP *or *B-FABP) *[3,4] was among the most significantly differentially expressed genes (unpublished results).

FABP7 belongs to a family of structurally-related proteins showing tissue-specific patterns of expression. Nine FABPs (FABP1 – FABP9), expressed in normal liver, intestine, heart, adipose tissue, epidermis, brain, peripheral nervous system and testis have been identified (reviewed in [5]). FABP proteins are involved in lipid metabolism, including uptake and intracellular trafficking of fatty acids and retinoids. In addition, they play a role in gene regulation, cell signaling, cell growth and differentiation [6].

Several reports have suggested a possible role for the different FABP proteins in cancer biology, linking their levels with either increasing or decreasing degree of malignancy. Adamson et al. reported that FABP5 (C-FABP/E-FABP) protein expression is higher in prostate cancer compared to prostatic hyperplasia [7]. On the other hand, the FABP1 level decreases with progression of colon cancer [8]. FABP7 is highly expressed in glia cells throughout development of the nervous system [4,9] and high FABP7 expression in glioblastomas is related to poor prognosis [10]. Recently, two studies have addressed FABP7 expression in surgical specimens from melanoma patients. While de Wit et al. reported down-regulation of FABP7 in melanomas compared to nevi, Goto et al. found FABP7 to be frequently expressed in melanomas, and suggested that it may play a role in cell proliferation and invasion[11,12].

In the current study we examined the role of FABP7 in proliferation, apoptosis and invasion of melanoma cells grown *in vitro *and studied possible regulation mechanisms of this protein. In addition, we examined the expression of FABP7 protein in clinical melanoma specimens and assessed the relationship between FABP7 expression pattern and known prognostic variables, cell cycle factors and disease progression. We report that FABP7 is regulated via PKC and the MAPK/ERK1/2 signaling pathway in melanoma cells *in vitro *and promotes proliferation and invasion. Moreover, FABP7 expression is associated with tumor thickness and proliferation in melanoma biopsies.

## Methods

### Cell lines and Growth Conditions

The Wistar Melanoma (WM) cell lines were kindly provided by Dr. Meenhard Herlyn (Wistar Institute, Philadelphia, PA) and have been described in detail elsewhere [13].

The MeWo cell line was derived from a lymph node metastasis [14]. The cell lines FEMX-I and LOX were established from metastatic lymph node biopsies obtained from melanoma patients treated at the Rikshospitalet-Radiumhospitalet Medical Center [15]. The cells were routinely cultured in RPMI 1640 medium (BioWhittaker Europe, Verviers, Belgium) supplemented with 5% fetal calf serum (FCS) (Biochrom, KG, Berlin, Germany). Phorbol-12-myristate-13-acetate (PMA) was from Sigma-Aldrich (St. Louis, MO), whereas the MEK1 inhibitor, PD98059, was from Cell Signaling Technology (Beverly, MA). Multi-cellular aggregates (spheroids) were prepared as previously described [16]. Briefly, 24-well plates were coated with 1% Seaplaque agarose (BioWhittaker Molecular Application, Rockland, ME) and tumor cells (2 × 10^5 ^cells in 1 ml complete medium) were plated on top of the solidified agarose. For thymidine incorporation assay, 5000 cells per well were plated in 96-well polyhema (Sigma-Aldrich)-coated U-bottom plates. For treatment of spheroid cultures, PMA was added when plating in suspension, whereas the inhibitors in combination experiments were added 45 min prior to plating as spheroids.

### Gene expression analysis

WM35 cells were grown as spheroids for 24 hrs in the presence of PMA and PD98059, alone and in combination. Total RNA was extracted using the TRIZOL reagent (Invitrogen, Carlsbad, CA). Gene expression profiling was performed using Affymetrix U133 Plus 2.0 arrays (Affymetrix, Santa Clara, CA). For microarray hybridization, the protocol described in the Affymetrix GeneChip eukaryotic one-cycle target preparation protocol, using 5 μg of total RNA, was followed. Analysis of the data was performed by Genolyze Ltd. (Turku, Finland) using statistical software R version 2.3.0. and package collection Bioconductor version 1.8. Statistical significance was assessed using p-value from two-tailed two sample t-test. P-values are replaced with q-values to control the False Discovery Rate.

### Quantitative real time RT-PCR analysis

The high capacity cDNA reverse transcription kit (Applied Biosystems, Foster city, CA) was used to reverse-transcribe total RNA (0.8 μg) in a 20 μl reaction mixture using random primers. The real-time PCR analyses were performed using TaqMan Fast Universal PCR Master Mix (2×) and TaqMan Gene Expression Assay (HS00361426-ml FABP7, HS99999908-ml GUS, Applied Biosystems). A total of 0.5 μl cDNA was used in 25 μl PCR mixtures with 900 nM of each primer and 250 nM TaqMan probe. The reactions were carried out in a 7900 HT Fast Real Time PCR system (Applied Biosystems) with the following program: 95°C for 20 sec. followed by 40 cycles of 95°C for 1 sec., 60°C for 20 sec. Each sample was run in triplicate. The *FABP7 *relative mRNA expression level was normalized with respect to the beta-glucuronidase (GUS) gene, which had stable transcript levels under these experimental conditions. The mean from three independent experiments was calculated.

### Immunoblotting

Cells were lysed in ice-cold NP-40 lysis buffer (1% NP-40, 10% glycerol, 20 mM Tris-HCl, pH 7.5, 137 mM NaCl, 100 mM sodium vanadate, 1 mM phenylmethylsulfonyl fluoride (PMSF), 0.02 mg/ml each of aprotinin, leupeptin, and pepstatin, and 10 μl/ml phosphatase inhibitor cocktail I and II (Sigma-Aldrich)). Protein quantitation was done by Bradford analysis and 25 μg protein/lane was resolved by SDS polyacrylamide gel electrophoresis. Transfer and hybridization were as described in [17]. To ensure even loading, filters were stained with naphthol-blue black (Sigma-Aldrich) and re-stained with α-tubulin. The antibodies against FABP7 and α-tubulin were from R&D Systems (Minneapolis, MN) and Calbiochem (San Diego, CA), respectively. HRP-conjugated anti-mouse IgG secondary antibody was from Promega (Madison, WI) and HRP-conjugated anti-goat secondary antibody was from DAKO A/S (Glostrup, Denmark).

### Small interfering RNA transfection

Fifty thousand cells per well were seeded in 24-well plates for 24 hrs prior to transfection with 50 nM siRNA targeting FABP7 (OligioID: HSS103516; Catalog# 1299003) or negative control siRNA duplexes (Catalog#12935-300) using Lipofectamine™ RNAiMAX transfection reagent (all reagents and siRNA were from Invitrogen). Cells were detached 48 hours after transfection and plated into agarose-coated 24-well plates as spheroids for an additional 72 hrs for assessment of apoptosis, seeded into 96-well polyhema-coated U-bottom plates for the proliferation assay and plated in BioCoat Matrigel invasion chambers.

### Proliferation assay

Five thousand cells per well were seeded in 96-well polyhema-coated U-bottom plates for spheroids and in 96-well flat-bottom plates for monolayer cells and cultured for 72 hrs, the last 24 hrs with the addition of 3.7 × 10^4 ^Bq [^3^H]Thymidine (ARC, St.Louis, MO) Thereafter, the cells were harvested using a Filtermate Harvester (Packard Instrument Co. Meriden, CT). [^3^H]Thymidine incorporation was assessed in a Packard Microplate Scintillation Counter. Proliferation assays were measured in triplicate. The experiment was repeated at least three times.

### Flow cytometric analysis of apoptosis

The adherent cells were harvested by Trypsin and together with detached cells fixated in 100% cold methanol. Fixed cells were washed with PBS, incubated for 30 min at 37°C in 50 μl terminal transferase (TdT) solution containing 5 units TdT (Roche, Basel, Switzerland), 10 μl 5× reaction buffer (supplied with TdT), 1.5 mM CoCl_2_, 0.5 nmol labeled biotin-16-dUTP, 0.1 mM dithiothreitol and distilled water. The cells were subsequently washed once in PBS containing 0.1% Triton X-100 and incubated in 50 μl 1:50 streptavidin-FITC (Amersham, Buckinghamshire, UK) in PBS (0.1% Triton X-100) and 3% skimmed dry milk for 45 min at room temperature. After washing in PBS (0.1% Triton X-100) the pellet was resuspended in PBS (0.1% Triton X-100) containing 2 μg/ml Hoechst 33258 to a final concentration of 1 × 10^6 ^cells/ml and incubated for 30 min at 4°C. Data acquisition and analysis were performed on Becton Dickinson LARII (Becton Dickinson immunocytometry systems, San Jose, CA) using Multifit software (FACSDiVa House inc., Tonsham, ME).

### Matrigel invasion assay

WM35 and WM239 cells were plated in BioCoat Matrigel invasion chambers (BD Biosciences, San Jose, CA) at a cell density of 3 × 10^4 ^per chamber in RPMI 1640 supplemented with 5% fetal bovine serum (inner chamber) 48 hrs post-transfection. Self-supplied fibroblast conditioned medium was used as chemoattractant in the outer chamber. The conditioned medium was obtained from fibroblasts isolated as described by Costea *et al*[18] cultivated in DMEM supplemented with 10% fetal bovine serum. The medium was collected when the cells were 70% confluent. After 48 hrs incubation at 37°C and 5% CO_2_, non-invading cells remaining on the top surface of the chamber were removed by scrubbing with a cotton-tipped swab, and the invading cells that had adhered to the bottom surface of the chamber membranes were fixed, stained with hematoxylin and counted.

### Clinical melanoma specimens

Formalin-fixed, paraffin-embedded tissue from 149 primary and 68 metastatic melanomas, as well as 11 benign nevi, was examined for expression of FABP7 protein. Of the primary tumors, 93 were classified as superficial spreading (SSM) and 56 as nodular melanomas (NM). Clinical follow-up was available for all patients. The study was approved by the Regional Committee for Medical Research Ethics in Norway.

### Immunohistochemical analysis

Sections of formalin-fixed, paraffin-embedded tissue were immunostained using the two-step EnVision system (DAKO EnVision™, DAKO A/S). Deparafinized sections were microwaved in low pH buffer (pH 6.0) (DAKO) at 750 W for 5 minutes and then at 500 W for 15 minutes to unmask the epitopes. After treatment with 1% hydrogen peroxide for 5 minutes to block endogenous peroxidase, the sections were incubated with polyclonal rabbit anti-human FABP7 antibody (R&D Systems) for 30 minutes at room temperature followed by 30 minutes incubation with mouse anti-goat antibody (Santa Cruz Biotechnology, Santa Cruz, CA). The sections were then incubated with HRP-labeled secondary antibody for 30 minutes followed by 5 minutes incubation at RT with DAB substrate (DAKO A/S). All series included positive controls. Four semiquantitative classes were used to describe the number of stained cells: negative, ≤ 5%, 6–50% and >50%. Both nuclear and cytoplasmic staining was scored. Staining was evaluated by a surgical pathologist (BD). A subset of the cases (n = 50) was additionally scored by another author (AS).

### Statistical analysis

Statistical analysis was performed using the SPSS program version 13.0 (Chicago, IL). The differences between FABP7 expression in benign nevi, primary melanomas and metastases were analyzed using the Chi-square test. The relationship between FABP7 expression and mean tumor thickness was evaluated nonparametrically using the Mann-Whitney two sample test. The association between expression of FABP7 and cell cycle markers was performed using the Fischer's exact test. Kaplan-Meyer estimates and the log-rank test were used for survival analysis. P < 0.05 was considered statistically significant.

## Results

### Identification of molecules involved in survival of melanoma cells as multicellular aggregates in suspension using gene expression profiling

We previously showed that PMA treatment protects melanoma cells from suspension-mediated apoptosis while the MEK1 inhibitor PD98059 has the opposite effect [2]. In order to identify new factors involved in anchorage-independent growth of melanoma cells, we compared mRNA expression profiles from the melanoma cell line WM35, cultured in monolayer or as untreated spheroids, as well as following treatment of the spheroids with PMA and/or PD98059 for 24 hours.

**Figure 1:**
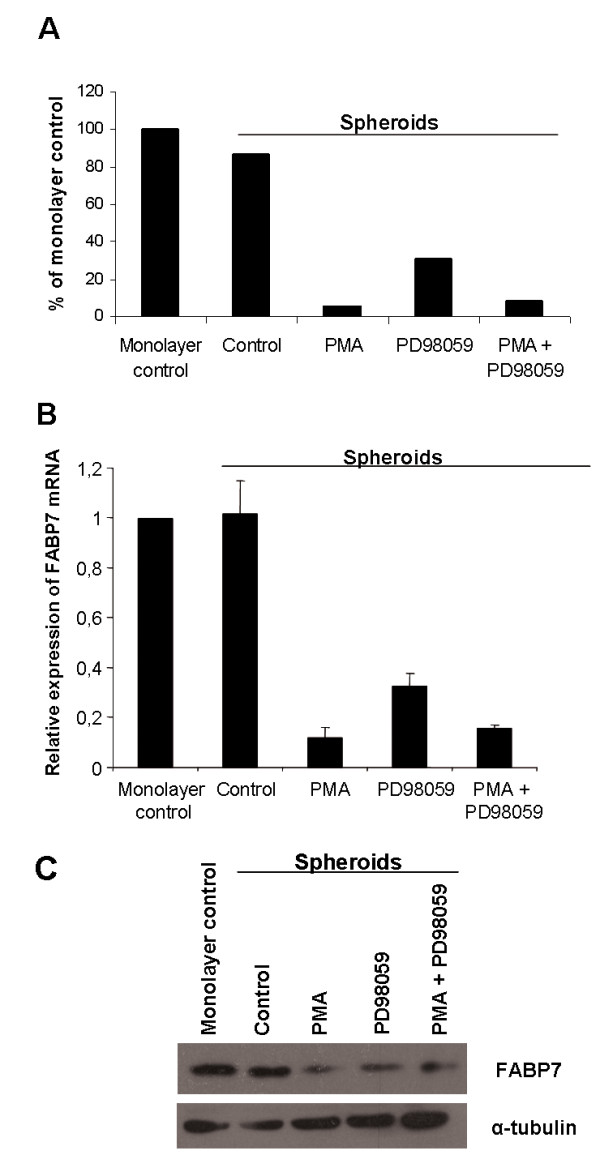
**A) The expression level of FABP7 as detected by Affymetrix microarray analysis in WM35 cells grown as spheroids for 24 hrs with or without PMA and/or PD98059 compared to untreated monolayer control.** B) *FABP7 *mRNA expression levels in the same cells as in (A) measured by real time RT-PCR. The results presented are relative to untreated monolayer control. The average was calculated from three independent experiments and presented with standard deviation. C) Expression of the FABP7 protein by Western blot analysis. Down-regulation of *FABP7 *mRNA and protein was seen after treatment with PMA and PD98059 for 24 hrs. α-tubulin was used as loading control.

The *FABP7 *gene was among the genes showing the highest differential expression. While no notable difference was observed between monolayer cells and spheroids, treatment with PMA or PD98059, as well as with PD98059 and PMA in combination, led to *FABP7 *mRNA down-regulation in treated spheroids compared to the spheroid control (Figure 1a). The microarray results were validated using real time RT-PCR (Figure 1b).

### FABP7 is expressed in melanoma cell lines and regulated through PKC and the MAPK/ERK1/2 signaling pathway

The protein level of FABP7 in monolayer culture, untreated spheroids and spheroids treated with PMA and/or PD98059 for 24 hrs was analyzed using western blot. As shown in Figure 1c, no change in FABP7 protein level was observed between monolayer cells and untreated spheroids while in spheroids treated with PMA and/or PD98059, the protein level was reduced compared to controls. This was in accordance with the reduction of FABP7 mRNA levels. A similar reduction in FABP7 protein level was obtained in monolayer cultures treated with PMA and/or PD98059 (data not shown).

**Figure 2:**
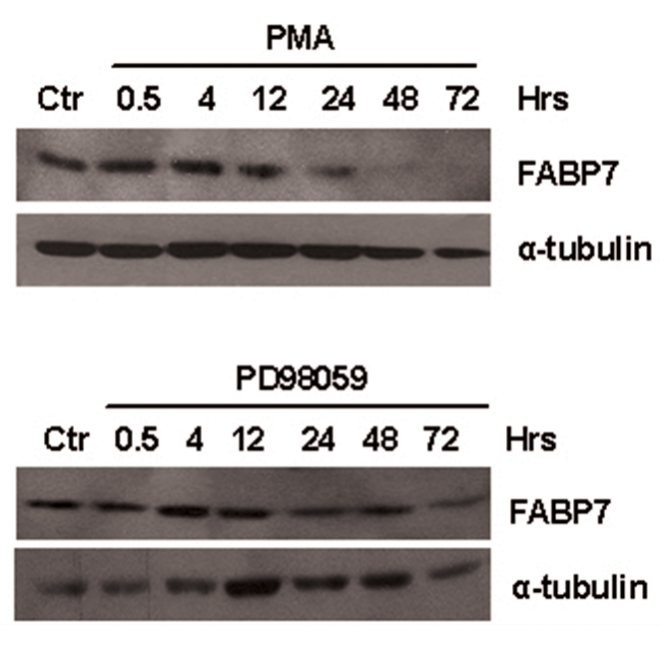
**Western blot showing the expression of the FABP7 protein in WM35 monolayer cells treated with PMA or PD98059 for 0.5 hrs, 4 hrs, 12 hrs, 24 hrs, 48 hrs and 72 hrs.** α-tubulin was used as loading control.

In order to reveal if FABP7 expression levels differ during the cultivation of the WM35 cells following PMA and/or PD98059 treatment, we performed a time course study. The monolayer cells were treated with PMA or PD98059 from 0,5 hrs to 72 hrs. As shown in Figure 2. we observed down-regulation of FABP7 protein after 12 hrs in both PMA and PD98059 treated cells although the effect of PMA was more pronounced over time. The down-regulation was sustained for up to 72 hrs for both treatments. These results were supported by real time RT-PCR (data not shown).

**Figure 3:**
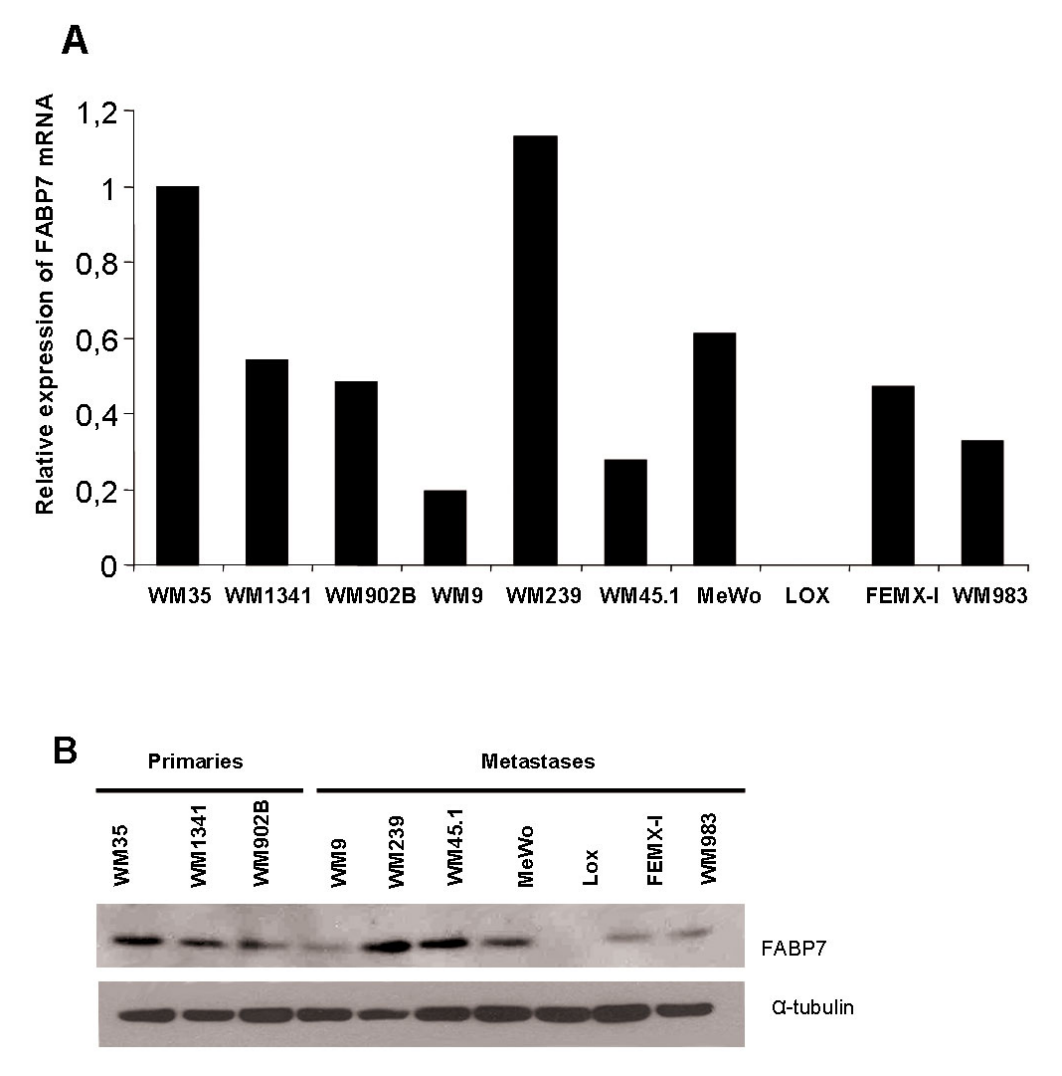
**A) *****FABP7***** mRNA level in melanoma cell lines as measured by real time RT-PCR analysis.** Three primary and 7 metastatic melanoma cell lines were evaluated. The expression levels are shown relative to the WM35 cell line. B) Expression of FABP7 protein by western blotting, with α-tubulin as loading control.

To examine if FABP7 is frequently expressed in melanoma cell lines we analyzed the level of FABP7 mRNA and protein in two primary (WM1341 and WM902B) and seven metastatic cell lines (WM239, WM45.1, WM983, WM9, LOX, MeWo and FEMX-I) in addition to WM35. As shown in Figure 3a and 3b, variable levels of FABP7 mRNA and protein were detected in 9 out of 10 cell lines. With the exception of WM45.1, good concordance between mRNA and protein levels was observed in all the cell lines. No clear differences were observed between FABP7 expression levels in cell lines originating from primary tumor vs. metastasis.

### FABP7 is involved in proliferation and invasion of melanoma cells

**Figure 4:**
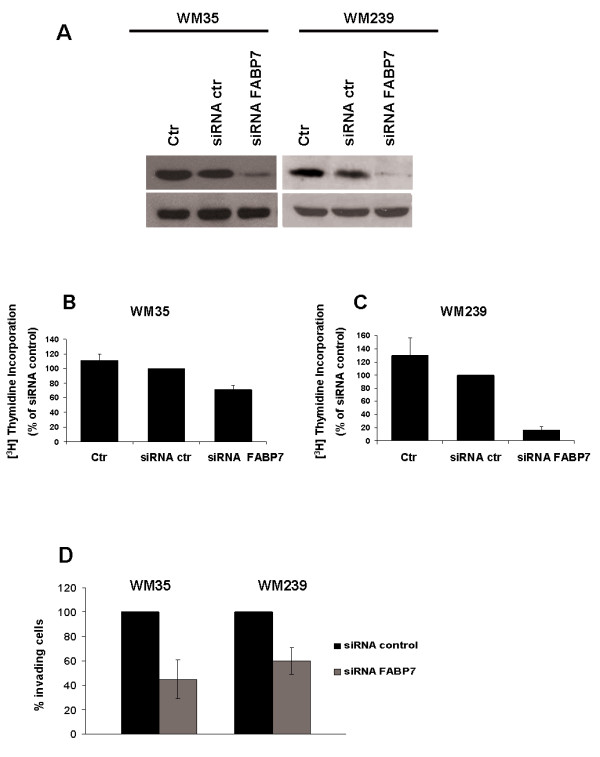
**A) Western blot analysis showing down-regulation of FABP7 in WM35 and WM239 cells after transfection with FABP7 siRNA, with α-tubulin as loading control.B-C) **The effect of FABP7 down-regulation on proliferation in WM35 (B)and WM239 (C)cells measured by [^3^H]Thymidine incorporation 72 hrs post transfection. Down-regulation of FABP7 protein using siRNA led to reduction of DNA synthesis, suggesting reduced proliferation in both cell lines.D) Matrigel invasion assay. Inhibition of invasion ability of WM35 and WM239 cells following FABP7 down-regulation with siRNA. The average was calculated from three independent experiments and presented with standard deviation.

In order to further investigate the function of FABP7 we chose to transiently down-regulate FABP7 using specific siRNA in the WM35 and WM239 cell lines, which we found to have high FABP7 expression. The effect of down-regulation on proliferation, invasion and apoptosis was examined. Monolayer cells were incubated for 48 hrs with FABP7 siRNA or a control siRNA and analyzed for transfection efficiency by western blot (Figure 4a). As demonstrated in figure 4b and 4c, FABP7 down-regulation reduced proliferation by 29% in WM35 and 84% in WM239 cells as compared to scrambled siRNA control transfected cells. Similar results were obtained when the cells were grown in suspension (data not shown).

The degree of apoptosis was assessed using TdT-mediated dUTP nick end labeling (TUNEL) staining and flow cytometry. Analysis of both monolayer and spheroid cultures showed that down-regulation of FABP7 did not affect the percentage of apoptotic cells (data not shown). Together these results suggest that FABP7 is most likely involved in proliferation and not apoptosis in melanoma cells.

We investigated the effect of *FABP7 *down-regulation on invasion using the Matrigel assay. The number of invading cells was reduced by 55% and 40% in WM35 and WM239 cell respectively after transfection with FABP7 siRNA compared with scrambled siRNA control-transfected cells (Figure 4d), suggesting that FABP7 contributes to the invasiveness of melanoma cells.

### FABP7 is expressed in melanomas and associated with tumor thickness

**Figure 5:**
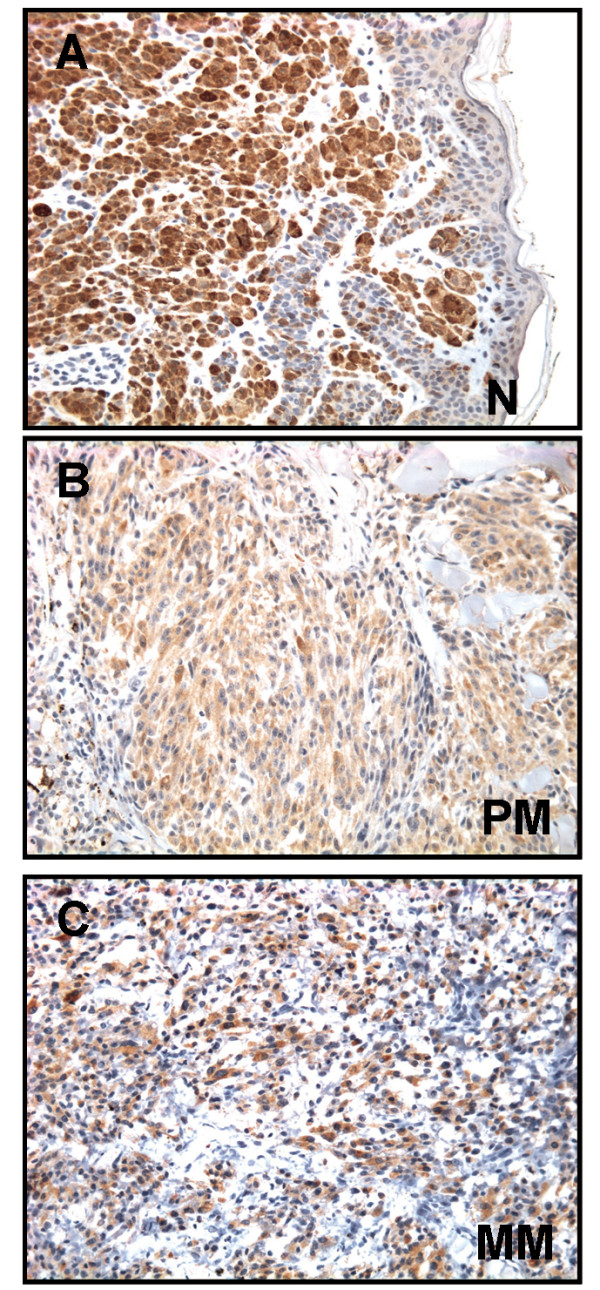
Immunohistochemical staining of FABP7 in a benign nevus **(A) **primary melanoma **(B) **and metastatic melanoma **(C)**.

In order to examine the clinical relevance of FABP7, paraffin-embedded tissue from a panel of benign nevi and primary and metastatic melanomas was analyzed for expression of FABP7 protein using immunohistochemistry. Heterogeneous cytoplasmic and/or nuclear expression of FABP7 was observed in 91% of the nevi, 71% of the primary tumors and 70% of the metastases. The results are summarized in Table 1 and 2 and illustrated in Figure 5. Statistical analysis demonstrated a significant higher cytoplasmic FABP7 expression in nevi compared to primary and metastatic melanomas (P = 0.023), with comparable nuclear expression. A two-tier analysis of primary and metastatic melanomas showed comparable expression for both cytoplasmic and nuclear expression (P > 0.05). Good concordance (>80%) was achieved between the two observers. Discrepant cases were resolved through a consensus session.

**Table 1:** Number (percentage) of melanocytic lesions expressing FABP7 in different cellular compartments

**Tumor Type**	**No. of tumors**	**Total no. of positive**	**Cytoplasm**	**Nucleus**	**Cytoplasm/nucleus**
**Benign nevi**	11	10 (91)	2 (18)	0 (0)	8 (73)
**Primary melanomas**	149	106 (71)	36 (24)	0 (0)	70 (47)
**SSM**	93	61 (66)	19 (21)	0 (0)	42 (45)
**NM**	56	45 (80)	17 (30)	0 (0)	28 (50)
**Metastases**	68	48 (70)	20 (29)	1 (2)	27 (39)

**Table 2:** Number (percentage) of melanocytic lesions expressing different levels of FABP7

**Tumor type**	**No. of tumors**	**Cytoplasm**				**Nucleus**			
		**-**	**≤ 5%**	**6–50%**	**> 50%**	**-**	**≤ 5%**	**6–50%**	**> 50%**

**Benign nevi**	11	1 (9)	0 (-)	1 (9)	9 (82)	3 (28)	4 (36)	4 (36)	0 (-)
**Primary melanomas**	149	43 (28)	14 (9)	42 (28)	50 (34)	79 (53)	51 (34)	16 (11)	3 (2)
**SSM**	93	32 (34)	10 (11)	25 (27)	26 (28)	51 (55)	31 (33)	9 (10)	2 (2)
**NM**	56	11 (20)	4 (7)	17 (30)	24 (43)	28 (50)	20 (36)	7 (12)	1 (2)
**Metastases**	68	21 (31)	8 (11)	21 (31)	18 (27)	40 (59)	20 (29)	7 (11)	1 (1)

**Figure 6:**
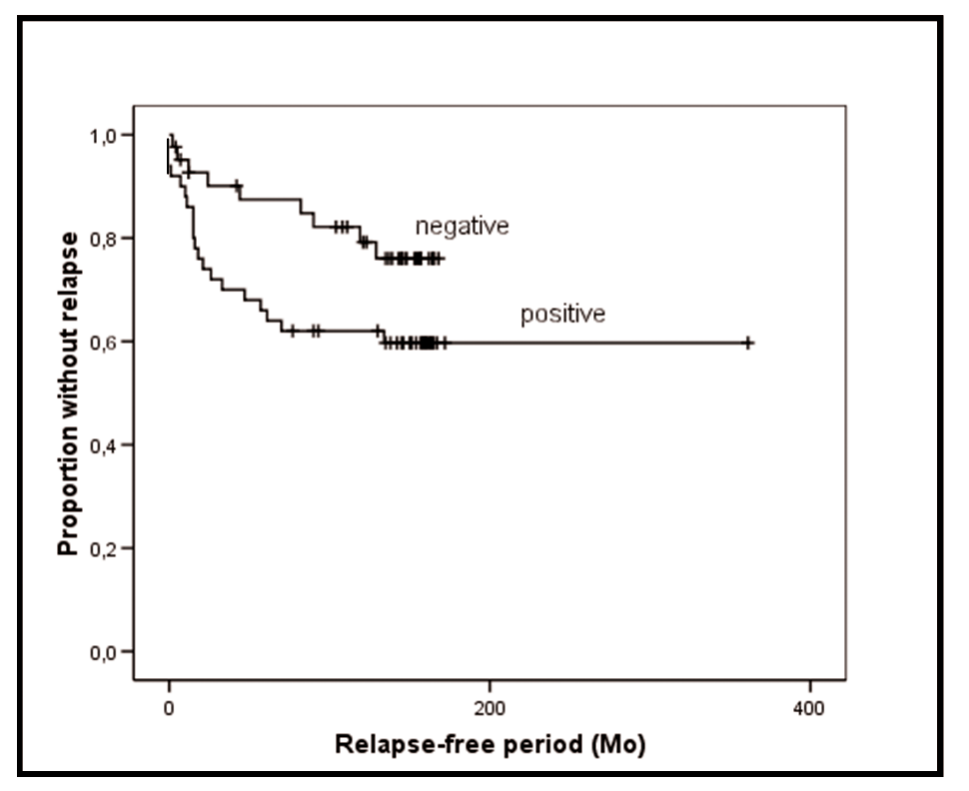
**Kaplan-Meier curve demonstrating a negative trend (*P *= 0.069) between protein expression of cytoplasmic FABP7 and relapse-free survival for patients with SSM.** FABP7 expression was considered high when > 5% of the tumor cells showed positive staining with the anti-FABP7 antibody.

Since 62% (92/149) of the primary tumors expressed cytoplasmic FABP7 in more than 5% of the cells, this cutoff was used to distinguish between high and low protein levels. Applying the same cutoff when evaluating nuclear staining, we observed that only 13% (19/149) of the tumors had high protein expression levels. Higher cytoplasmic FABP7 was significantly associated with increased thickness of SSM (P = 0.021). In addition, in this group of patients, a trend towards increased relapse-free survival (P = 0.069) for patients whose tumors expressed less FABP7 was observed. No such correlation was observed in NM (Table 3 and Figure 6). We did not observe any significant correlation between FABP7 staining and overall survival for patients diagnosed with either SSM or NM (data not shown). Nuclear staining had no association with disease outcome (data not shown).

**Table 3:** Relationship between cytoplasmic FABP7 expression and tumor thickness in primary melanomas

**Tumor type**	**Expression**	**No of patients**	**Average depth of growth (mm)**	***P***
**SSM**	Negative*	41	1.59	
	Positive	48	2.17	0.021

**NM**	Negative*	15	4.32	
	Positive	41	4.78	0.697

* Considered negative if < 5% tumor cells showed positive immunoreactivety of FABP7

### Relationship between FABP7 expression and markers of proliferation

Since our panel of primary and metastatic melanomas has previously been analyzed for expression of cell cycle progression markers [19-21] and activation status of MAPK/ERK1/2 [22], it was of interest to examine the relationship between FABP7 expression and the levels of these factors. The results showed no correlation between cytoplasmic FABP7 expression and the expression of the cyclins A, D1 or D3 or the cdk inhibitors p21^CIP1/WAF1^and p27^Kip1 ^in either SSM or NM. However, a trend for an association between cytoplasmic FABP7 and Ki-67 (P= 0.07) in SSM was observed, which is in support of our *in vitro *results, suggesting FABP7 involvement in proliferation. Furthermore, expression of activated MAPK/ERK1/2 did not correlate with FABP7 expression. Interestingly, however, we observed that if the cutoff level was changed (only total lack of FABP7 was regarded as low expression), MAPK/ERK1/2 expression did positively correlate with cytoplasmic FABP7 expression in SSM (P= 0.047). This was not the case for NM, regardless of cutoff. Nuclear expression of FABP7 did not correlate with any of the examined markers (data not shown).

## Discussion

We previously showed that PMA-mediated PKC activation and activation of the MAPK/ERK1/2 pathway contributes to increased proliferation and reduced apoptosis of melanoma cells under anchorage-deprived conditions. In the present study we used gene expression profiling to identify additional genes involved in these processes, and found the *FABP7 *gene to be differentially expressed and down-regulated in WM35 spheroids following both PKC activation and MEK1 inhibition while no differences were observed between monolayer cells and untreated spheroids. PKC activation and MAPK/ERK1/2 down-regulation had opposite effect on anchorage-independent survival of the melanoma cells, but both negatively regulated FABP7. This observation argues against FABP7 involvement in promotion of anchorage-independent survival in these cells. Thus, it is likely that these pathways are regulating additional factors important for survival, independent of FABP7 down-regulation.

PKC is a well known activator of the MAPK/ERK1/2 pathway [23,24] and we have previously reported that PMA activates MAPK/ERK1/2 independently of its upstream activator MEK1 [2]. Since PMA treatment down-regulates FABP7 even in the presence of activated ERK1/2 this down-regulation is likely to be PMA/PKC-mediated but MAPK/ERK1/2-independent. Together this suggests that FABP7 can be regulated by both signaling pathways independently in melanoma cells. Several reports have shown that activation of the MAPK/ERK1/2 pathway can induce increased activity of peroxisome proliferator-activated receptors α/γ (PPAR α/γ). Similarly, PKC can both positively and negatively regulate PPARα-dependent transcription [25-27]. Binding of PPARγ to its response element, PPRE, has been shown to up-regulate FABP1 and FABP4 [5,28,29]. It is reasonable, therefore, to assume that FABP7 might also be regulated through this mechanism.

To further clarify the role of FABP7 in melanomas we used siRNA to down-regulate its expression in the primary WM35 and metastatic WM239 melanoma cell lines. This down-regulation notably inhibited proliferation in both cell lines, but did not affect the degree of apoptosis, arguing for involvement of FABP7 in melanoma proliferation. In support of our results, Goto *et al *[12] showed that proliferation of melanoma cell lines is reduced upon down-regulation of FABP7, also without affecting apoptosis. Our results showed that down-regulation of FABP7 negatively influences the invasive potential of melanoma cells, also in agreement with Goto *et al *[12] who demonstrated that down-regulation of FABP7 decreased invasiveness in 2 of 6 melanoma cell lines. In further support of this hypothesis are the data of Mita *et al*., who showed that FABP7 increases the invasion properties of astrocytoma cells [30]. Of note, when FABP7 was reintroduced in the metastatic cell line LOX, lacking constitutive FABP7 expression, no effect on apoptosis, proliferation or invasion was observed (preliminary results, data not shown). Similar results were reported by Goto *et al*. [12] in 4 out of 6 melanoma cell lines. However, the reason for the discrepancy between the cell lines is still unclear. Thus, the biological role and detailed functional mechanism of the FABP7 protein in melanoma cells remains to be further investigated.

Several members of the FABP family have been reported to be differentially expressed in cancer. Loss of expression of FABP4 was reported in bladder cancer while FABP1 and FABP2 are over-expressed in prostate and breast cancers [7,31-33]. In accordance with Goto *et al*. [12] we found that FABP7 is expressed in both primary and metastatic melanoma cell lines, as well as in melanocytic lesions. However, there were no clear differences in FABP7 expression levels in primary derived compared to metastatic derived cell lines, suggesting that FABP7 is not associated with tumor aggressiveness. On the other side, cell lines are cultured in artificial environments that can not be directly compared to tumors *in vivo *and a connection to tumor aggressiveness and progression can not be completely excluded. In support of this, analysis of the clinical data showed that thicker SSM expressed higher levels of FABP7. Furthermore, a trend between high levels of FABP7 and reduced disease-free survival for these patients suggest that FABP7 could contribute to disease progression, possibly by increasing the invasion potential of the tumors. In support of our results, a negative association between FABP7 expression and survival was recently observed for patients with glioblastoma [10,34]. We also observed a positive trend between FABP7 and the proliferation marker Ki-67 in SSM, suggesting that FABP7 may contribute to increased proliferation *in vivo*. Since the patient subgroups in the analyses were small, the suggested clinical significance of FABP7 expression remains to be confirmed in larger patient cohorts.

In the clinical specimens, FABP7 protein expression was highest in nevi, with no observed differences between primary and metastatic melanoma. This is in accordance with the study by de Wit *et al*. [11] who reported that FABP7 is down-regulated in melanoma tissue compared to nevi using oligonucleotide arrays. The higher expression of FABP7 in nevi compared to melanomas seems contradictory to the *in vitro *data in the present study, as well as to the association with clinical parameters of disease progression. We are unable to explain this discrepancy at present. However, the majority of benign nevi are terminal lesions that do not progress to melanoma and the molecular events regulating these processes might differ. It is also possible that different expression levels of FABP7 mediate different effects during disease progression.

Variation in sub-cellular localization of FABP7 has been reported in developing radial glia cells, glioma cell lines [9,35] and glioblastoma multiforme (GBM) specimens [34]. Since FABP proteins are considered to be co-activators in PPAR-mediated gene transcription control, this could in part explain FABP7 translocation to the nucleus (reviewed in [5]). Recently, it was reported that nuclear expression of FABP7 is restricted to infiltrative tumor types and related to EGFR amplification and over-expression as well as poor prognosis of GBM [10,34]. In our melanoma cohort we did not find any association between nuclear expression of FABP7 and disease-free or overall survival.

## Conclusion

We confirmed that FABP7 protein is expressed in melanocytic lesions and showed that it can regulate proliferation and invasion in melanoma cells *in vitro*. Our results further suggest that FABP7 can be regulated by PKC and the MAPK/ERK1/2 pathway through independent mechanisms. In addition, FABP7 expression is associated with proliferation and tumor thickness in the patients with SSM, suggesting that for these patients FABP7 could be a potential target for therapy.

## Competing interests

The authors declare that they have no competing interests.

## Authors' contributions

AS carried out the siRNA transfections, invasion experiments and evaluated immunohistochemical staining and drafted the manuscript. KJ performed immunohistochemistry, flow cytometry, and contributed to drafting the manuscript. MS and AKRR carried out optimalization of the real-time PCR and antibodies used in western blotting in the study. MS also performed technical phase of microarray experiment. BD evaluated immunohistochemical staining. GT participated in the design of the study and microarray analysis. VAF conceived of the study, and participated in its design and coordination and helped to draft the manuscript. All authors read and approved the final manuscript.

## Pre-publication history

The pre-publication history for this paper can be accessed here:


